# Effect of Yiqi Buxue Decoction on Hemodynamic Changes of the Uterine Artery and Fetal Umbilical Artery and Pregnancy Outcomes in Pregnant Patients with Pulmonary Arterial Hypertension

**DOI:** 10.1155/2021/1849114

**Published:** 2021-08-17

**Authors:** Rendong Han, Liya Gao, Hongbo Sun, Mei Li, Chunxia Deng

**Affiliations:** ^1^Department of Obstetrics, Affiliated Hospital of Qingdao University, Qingdao 266003, Shandong Province, China; ^2^Department of Gynecology and Obstetrics, Beijing ChuiYangLiu Hospital, Beijing 100022, China

## Abstract

**Objective:**

To explore the effect of Yiqi Buxue decoction on hemodynamic changes of the uterine artery and fetal umbilical artery and pregnancy outcomes in pregnant patients with pulmonary arterial hypertension (PAH).

**Methods:**

120 pregnant patients with PAH treated in our hospital (January 2019-January 2020) were chosen as the research objects, and randomly split into group A (*n* = 60) and group B (*n* = 60). Both groups received routine treatment, and group B was treated with sildenafil citrate, while group A was treated with Yiqi Buxue decoction combined with sildenafil citrate. Both groups received 6 weeks of treatment to analyze the hemodynamic changes of the uterine artery and fetal umbilical artery and compare the cardiopulmonary function indexes and pregnancy outcomes between the two groups.

**Results:**

The hemodynamic indexes of the uterine artery and fetal umbilical artery, cardiopulmonary function indexes, and pregnancy outcomes in group A after treatment were notably better compared with group B (*P* < 0.01).

**Conclusion:**

Yiqi Buxue decoction can stabilize the hemodynamics of pregnant patients with PAH, improve their cardiopulmonary function, alleviate hypotension, and thus, reduce the possibility of adverse pregnancy outcomes, which should be popularized in practice.

## 1. Introduction

Pulmonary hypertension (PAH) during pregnancy refers to structural and functional changes in the pulmonary vessels caused by nonspecific causes, which is mainly characterized by elevated pulmonary vascular resistance (PVR). With obvious hemodynamic abnormalities and significantly increased cardiac load, patients are prone to heart failure under the influence of physiological changes during pregnancy, which seriously threatens maternal and child health [1–3]. Studies have shown that PAH is one of the leading important causes of perinatal death in patients, accounting for 12% of all causes of maternal death. With the continuous optimization of medical technology in recent years, the perinatal mortality rate of PAH has decreased from 33%–42% to 4.2%–15.0% [4], indicating an increased survival rate of patients. However, for patients with Eisenmenger syndrome, the mortality rate remains about 25% [5, 6], suggesting a high possibility of adverse pregnancy outcomes. At present, Western medicine treatment is often adopted to improve the hemodynamics of patients in practice, and targeted PAH drugs are gradually introduced, including anticoagulant drugs such as sildenafil citrate to alleviate the hypercoagulable state of patients and enhance their cardiopulmonary function. However, most PAH drugs are expensive [7] and cannot be widely applied in the early stage of the disease, so it is extremely important to choose cheaper and more efficient drugs for treatment. Yiqi Buxue decoction is a common drug to regulate qi and blood in clinic, which is able to improve the obstructed blood flow of patients, reduce the vascular resistance, enhance the cardiopulmonary function, optimize the microcirculation, enhance the antihypoxia ability of tissue, and stabilize the patients' internal environment. However, no research on the application of Yiqi Buxue decoction in PAH has been reported in academia. Based on this, Yiqi Buxue decoction was adopted in this paper for adjuvant treatment of PAH patients, summarized as follows.

## 2. Materials and Methods

### 2.1. General Information

The study is a randomized controlled trial. One hundred and twenty pregnant patients with PAH treated in Beijing ChuiYangLiu Hospital (January 2019-January 2020) were chosen as the research objects and randomly split into group A (*n* = 60) and group B (*n* = 60). No statistical difference was found in general information between the two groups (*P* > 0.05; Table 1). This study was approved by the hospital ethics committee, and all experiments conformed to the provisions of the Declaration of Helsinki.

### 2.2. Inclusion Criteria

The inclusion criteria of this study were as follows. (1) The patients or their families fully recognized the study process and signed the informed consent; (2) the patients were diagnosed with PAH during pregnancy after examination, according to the diagnostic criteria of *ESC/ERS Pulmonary Hypertension Guidelines* [8, 9]; and (3) the patients did not receive relevant treatment before enrollment [10, 11].

### 2.3. Exclusion Criteria

The exclusion criteria for patients in this study were as follows. (1) The patients had mental problems or could not communicate with others; (2) the patients had other organic diseases; (3) the patients were complicated with preeclampsia, endocrine system diseases, and other diseases; (4) the patients had chronic hypertension; and (5) the patients had placenta previa, or the fetuses had congenital malformation.

## 3. Methods

The patients in both groups received routine treatment, including introducing a low-salt and healthy diet, performing oxygen inhalation and cardiotonic treatment, limiting their physical activity of the patients, and arranging reasonable daily work for them. In addition, group B was treated with sildenafil citrate, while group A was treated with Yiqi Buxue decoction combined with sildenafil citrate. Both groups received 6 weeks of treatment, specifically as follows. (1) Sildenafil citrate: the patients took sildenafil citrate daily (Guangzhou Baiyunshan Pharmaceutical Holdings Co., Ltd., Baiyunshan Pharmaceutical General Factory; NMPA approval no. H20143255) before meals, 3 times a day and 25 mg each time. (2) Yiqi Buxue decoction: the decoction included 15 g of *Codonopsis pilosula*, 15 g of *Astragalus membranaceus*, 10 g of *Angelica sinensis*, 10 g of *Ligusticum wallichii*, 10 g of Radix Paeoniae Alba, 10 g of prepared *Rehmannia* root, 10 g of *Poria cocos*, 10 g of Chinese yam, 10 g of motherwort, and 5 g of liquorice. The drugs were decocted in warm water, and the decoction was taken with one dose a day after breakfast and dinner.

### 3.1. Observation Criteria

1. Hemodynamic changes of the uterine artery and fetal umbilical artery: all patients were examined by using a color Doppler ultrasound (Color ultrasound diagnostic instrument Voluson P6 produced by GE Healthcare; NMPA certified no. 20152062178) before and after treatment. With the probe frequency of 3 MHz and pulse sampling volume of 2 mm, the two-dimensional ultrasound diagnostic instrument was placed at the distal branch of the internal iliac artery to position the uterine artery and at 4 cm from the placenta to position the umbilical artery. Three stable arterial blood flow spectra were continuously measured to record the pulsatility index (PI), resistance index (RI), and the ratio of peak systolic velocity (*S*) to end-diastolic peak velocity (*D*) of the uterine artery and fetal umbilical artery.
2. Cardiopulmonary function indexes: fasting arterial blood and central venous blood were collected before and after treatment. Arterial oxygen saturation and arterial oxygen partial pressure were measured. The D-dimer level was detected by immunoturbidimetry (kits: Nanjing Getein Biotechnology Co., Ltd., Jiangsu Medical Products Administration No. 2400146). The level of N-terminal prohormone of brain natriuretic peptide (NT-proBNP) was detected by radioimmunoassay (Cobase 411 electrochemiluminescence instrument with matching reagent; NMPA certified no. 3402843).
3. Pregnancy outcomes: pregnancy outcomes included fetal pregnancy outcomes and patient pregnancy outcomes. The number of adverse pregnancy outcomes was recorded.

### 3.2. Statistical Treatment

In this study, the data were processed by SPSS20.0 software and graphed by GraphPad Prism 7 (GraphPad Software, San Diego, USA). The data included enumeration data and measurement data, tested by the *X*^2^ and *t*-test. The differences were statistically significant when *P* < 0.05.

## 4. Results

### 4.1. Comparison of Hemodynamic Changes of the Uterine Artery and Fetal Umbilical Artery

**Figure 1:**
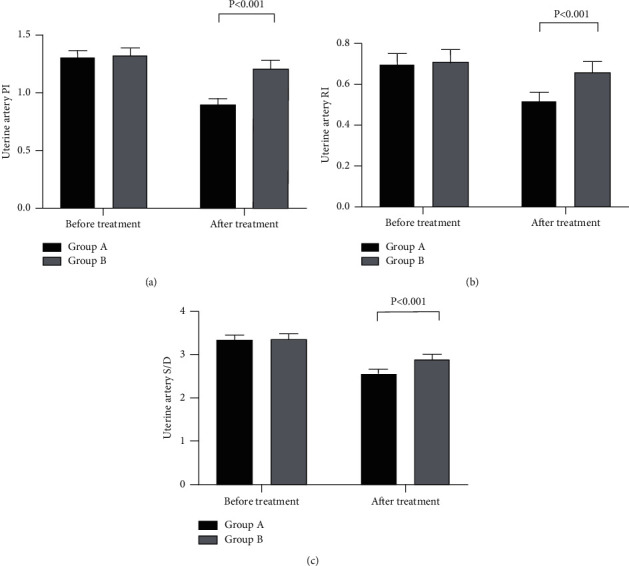
Comparison of hemodynamic changes in the uterine artery ($\bar{x}$ ± *s*). The abscissa represents before and after treatment. The black area is group A, and the gray area is group B. (a) The uterine artery PI. With no statistical difference in the PI between the two groups before treatment (1.31 ± 0.06 and 1.32 ± 0.07, *P* > 0.05), the PI in group A after treatment was notably lower compared with that in group B (0.90 ± 0.05 and 1.21 ± 0.07, *P* < 0.001). (b) The uterine artery RI. With no statistical difference in the RI between the two groups before treatment (0.70 ± 0.05 and 0.71 ± 0.06, *P* > 0.05), the RI in group A after treatment was notably lower compared with that in group B (0.52 ± 0.04 and 0.66 ± 0.05, *P* < 0.001). (c) Uterine artery *S*/*D*. With no statistical difference in the *S*/*D* between the two groups before treatment (3.35 ± 0.10 and 3.37 ± 0.12; *P* > 0.05), the *S*/*D* in group A after treatment was notably lower compared with group B (2.56 ± 0.11 and 2.89 ± 0.12, *P* < 0.001).

**Figure 2:**
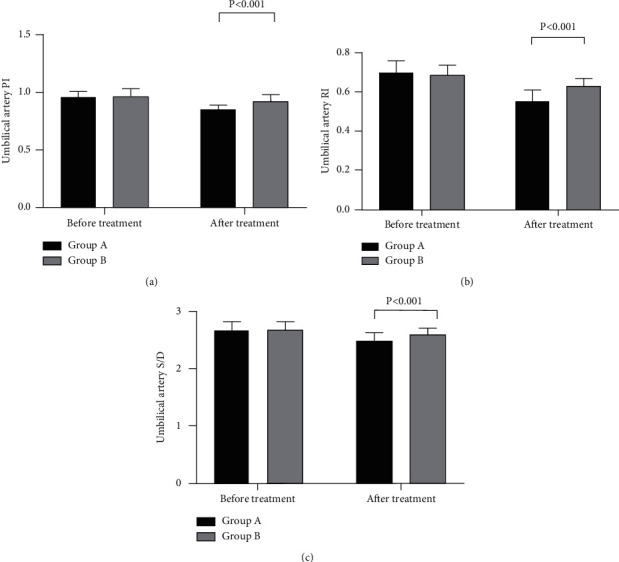
Comparison of hemodynamic changes in the fetal umbilical artery ($\bar{x}$ ± *s*). The abscissa represents before and after treatment. The black area is group A, and the gray area is group B. (a) The umbilical artery PI. With no statistical difference in the PI between the two groups before treatment (0.96 ± 0.05 and 0.97 ± 0.06, *P* > 0.05), the PI in group A after treatment was notably lower compared with that in group B (0.85 ± 0.04 and 0.92 ± 0.06, *P* < 0.001). (b) The umbilical artery RI. With no statistical difference in the RI between the two groups before treatment (0.70 ± 0.06 and 0.69 ± 0.05, *P* > 0.05), the RI in group A after treatment was notably lower compared with that in group B (0.55 ± 0.06 and 0.63 ± 0.04, *P* < 0.001). (c) Umbilical artery *S*/*D*. With no statistical difference in the *S*/*D* between the two groups before treatment (2.67 ± 0.15 and 2.68 ± 0.14, *P* > 0.05), the *S*/*D* in group A after treatment was notably lower compared with that in group B (2.48 ± 0.15 and 2.59 ± 0.12, *P* < 0.001).

After treatment, the hemodynamics indexes of the uterine artery and fetal umbilical artery in group A were notably better compared with those in group B (*P* < 0.001; Figures 1 and 2).

### 4.2. Comparison of Cardiopulmonary Function Indexes

The cardiopulmonary function indexes of group A after treatment were notably better compared with those of group B (*P* < 0.001; Table 2).

### 4.3. Comparison of Pregnancy Outcomes

The pregnancy outcomes in group A were notably better compared with those in group B (*P* < 0.001; Table 3).

## 5. Discussion

Occurring mostly in women of childbearing age, PAH will cause intimal hyperplasia of the pulmonary arteries, autochthonous thrombus, and increased pulmonary vascular resistance, ultimately increasing the right heart load of patients and leading to heart failure or death in severe cases. Both secondary PAH and primary PAH are absolute contraindications of pregnancy because patients in pregnancy will undergo a series of physiological changes that will greatly increase the mortality and the incidence of adverse pregnancy outcomes [6, 12–14]. For the patients, changes during pregnancy include increased blood volume, physiological anemia, the abnormal cardiac output that can increase by about 50%, and a hypercoagulable state in the body of patients. In the third trimester, the returned blood volume of patients significantly decreases, significantly increasing the possibility of heart failure or death [15–17]. For the fetuses, PAH can reduce the placental blood perfusion. Imaging examination often shows increased resistance of the uterine artery and umbilical artery, so the fetuses cannot obtain nutrition from the placenta and suffer from long-term chronic hypoxia with the mothers, which seriously affects their life health. In this study, the results showed that the hemodynamics indexes of uterine artery and fetal umbilical artery in group A were notably better compared with those in group B after treatment. Yiqi Buxue decoction has been found to have the function to resist platelet aggregation and reduce vascular resistance [18]. Therefore, it suggested that Yiqi Buxue decoction could effectively improve the hemodynamics of the patient.

Due to the high risk of pregnant patients with PAH, clinical practice has been promoting contraception knowledge of PAH women, while improving PAH treatment methods [19]. Since 1990, some progress has been made in the treatment of PAH in China, and the mortality rate of patients with mild PAH has currently decreased to less than 10%, but the prognosis of patients with severe PAH remains unsatisfactory [20]. Although studies have shown that medication from early pregnancy can optimize the microcirculation of patients, drugs such as endothelin antagonists leave adverse effects on mothers and fetuses and cannot effectively improve maternal and fetal outcomes [21]. Sildenafil citrate selected in this study is a drug to treat PAH with high safety, which can reduce the hypoxemia of patients and optimize their cardiopulmonary function. Therefore, the results showed that the cardiopulmonary indexes and oxygen saturation of both groups were improved, indicating that the maternal and fetal hypoxia was alleviated. Based on this, group A was additionally treated with Yiqi Buxue decoction that contained *Astragalus membranaceus*, *Angelica sinensis*, *Poria cocos*, motherwort, and *Ligusticum wallichii*. Among them, *Astragalus membranaceus* can resist platelet aggregation, reduce vascular resistance, enhance the pulmonary ventilation function, and boost the cardiopulmonary function of patients, thus improving their oxygen utilization and antihypoxia ability [22, 23]. *Angelica sinensis* can alleviate tissue damage caused by ischemia, reduce blood viscosity, and play a positive role in replenishing qi and enriching the blood. *Poria cocos* and motherwort promote blood circulation to remove blood stasis, with the effects of detumescence and diuresis. Therefore, group A after treatment had a lower D-dimer level and notably better microcirculation compared with group B. This demonstrated that the hemodynamic changes of the uterine artery and umbilical artery in group A were better, so the fetuses could obtain a stable source of nutrients, thus reducing the chance of low-birth-weight newborns. Zhu et al. have found that ligustrazine not only has a good effect of dilating arteries and reducing the pulmonary arterial pressure of PAH patients but also has an antioxidant effect to scavenge oxygen free radicals and reduce the NT-proBNP level [24]. NT-proBNP, a common evaluation index of cardiac function in clinic, is positively correlated with pulmonary arterial pressure, so the decreased NT-proBNP indicated a lower degree of PAH in patients. Therefore, the lower NT-proBNP in group A represented more obvious improvement of PAH and a lower risk of patients during pregnancy. In addition, *Ligusticum wallichii* can alleviate pulmonary arteriospasm and further increase the placental blood supply and oxygen transportation by optimizing the blood circulation, making the fetuses less likely to suffer from intrauterine distress or asphyxia and comprehensively reducing the adverse effect of PAH on the fetuses.

The results showed that Yiqi Buxue decoction could be taken in the early pregnancy to reduce the negative effect of physiological changes during pregnancy on PAH patients. However, the patients were not grouped in detail according to the gestational age in this study. Whether the patients can take Yiqi Buxue decoction alone, whether they can take it throughout the pregnancy, and its effect on different gestational weeks still need to be further explored.

In conclusion, Yiqi Buxue decoction can stabilize the hemodynamics of pregnant patients with PAH, improve their cardiopulmonary function, alleviate hypotension, and thus, reduce the possibility of adverse pregnancy outcomes, which should be popularized in practice.

## Figures and Tables

**Table 1:** Comparison of general data of patients.

Items	Group A (*n* = 60)	Group B (*n* = 60)	*X*^2^/*t*	*P*
Age (years)				
Range	20–45	21–45		
Average age	32.98 ± 5.68	32.15 ± 5.98	0.780	0.437

Gestational age (weeks)				
Range	6–37	6–38		
Average gestational age	33.26 ± 5.98	33.45 ± 5.24	0.185	0.854
Number of multiparae	21	20	0.037	0.847
Number of primiparae	39	40		

Causes of PAH				
Congenital heart disease (CHD)	25	24	0.035	0.853
Rheumatic heart disease (RHD)	15	16	0.044	0.835
Hypertensive heart disease (HHD)	10	12	0.223	0.637
Other	10	8	0.261	0.609
Number of fetus			0.261	0.609
Single birth	50	52		
Multiple birth	10	8		
Number of patients who received cardiac therapy	2	3	0.209	0.648
Residence			0.040	0.841
Urban area	42	43		
Rural area	18	17		
Monthly income (yuan)			0.035	0.852
≥4000	36	37		
＜4000	24	23		

PAH degree				
Mild	38	40	0.147	0.702
Moderate	16	15	0.044	0.835
Severe	6	5	0.100	0.752
Education			0.139	0.709
Senior high school and below	25	23		
University and above	35	37		

**Table 2:** Comparison of cardiopulmonary function indexes ($\bar{x}$ ± *s*).

Indexes	Group A		Group B		*t*	*P*
Oxygen saturation (%)	Before treatment	85.45 ± 5.98	Before treatment	84.98 ± 5.45	0.450	0.654
	After treatment	97.68 ± 5.41	After treatment	90.22 ± 5.24	7.672	<0.001
	*t*	11.748	*t*	5.369		
	*P*	<0.001	*P*	<0.001		

Arterial oxygen partial pressure (mmHg)	Before treatment	49.21 ± 4.23	Before treatment	49.36 ± 4.25	0.194	0.847
	After treatment	62.65 ± 4.25	After treatment	53.65 ± 4.21	11.654	<0.001
	*t*	17.362	*t*	5.555		
	*P*	<0.001	*P*	<0.001		

D-dimer (mg/L)	Before treatment	2.05 ± 0.15	Before treatment	2.06 ± 0.17	0.342	0.733
	After treatment	1.78 ± 0.12	After treatment	1.89 ± 0.14	4.621	<0.001
	*t*	10.887	*t*	5.979		
	*P*	<0.001	*P*	<0.001		

NT-proBNP (pg/ml)	Before treatment	1100.65 ± 150.56	Before treatment	1115.65 ± 149.65	0.547	0.585
	After treatment	342.58 ± 40.68	After treatment	500.15 ± 50.23	18.883	<0.001
	*t*	37.651	*t*	30.203		
	*P*	<0.001	*P*	<0.001		

**Table 3:** Comparison of pregnancy outcomes [*n* (%)].

Outcomes	Group A (*n* = 60)	Group B (*n* = 60)	*X* ^2^	*P*
Fetal pregnancy outcomes				
Low birth weight	3 (5.0)	7 (11.7)	1.746	0.186
Neonatal asphyxia	5 (8.3)	10 (16.7)	1.905	0.168
Premature delivery	6 (10.0)	10 (16.7)	1.154	0.283
Death	2 (3.3)	5 (8.3)	1.365	0.243
Fetal distress	3 (5.0)	10 (16.7)	4.227	0.040
Premature rupture of membranes	5 (8.3)	6 (10.0)	0.100	0.752
Total	24 (40.0)	48 (80.0)	20.000	<0.001

Patient pregnancy outcomes				
Polyhydramnios	2 (3.3)	8 (13.3)	3.927	0.048
Postpartum hemorrhage	5 (8.3)	8 (13.3)	0.776	0.378
Death	0 (0.0)	2 (3.3)	2.034	0.154
Caesarean section	20 (33.3)	30 (50.0)	3.429	0.064
Total	27 (45.0)	48 (80.0)	15.680	<0.001

## Data Availability

All data can be obtained from the corresponding authors.
